# Impact of an Educational Intervention Aimed at Nursing Staff on Oral Hygiene Care on the Incidence of Ventilator-Associated Pneumonia in Adults Ventilated in Intensive Care Unit

**DOI:** 10.17533/udea.iee.v39n3e06

**Published:** 2021-11-08

**Authors:** Melissa Sánchez Peña, Luz Angélica Orozco Restrepo, Freddy Andrés Barrios Arroyave, Oscar Felipe Suárez Brochero

**Affiliations:** 1 Odontologist, Health Management and Audit Specialist. Fundación Universitaria Autónoma de las Américas, Pereira, Colombia Email: melissa.sanchez@uam.edu.co Fundación Universitaria Autónoma de las Américas Fundación Universitaria Autónoma de las Américas Pereira Colombia melissa.sanchez@uam.edu.co; 2 Nurse, M.Sc. Fundación Universitaria Autónoma de las Américas, Pereira, Colombia Email: luz.orozcor@uam.edu.co Fundación Universitaria Autónoma de las Américas Fundación Universitaria Autónoma de las Américas Pereira Colombia luz.orozcor@uam.edu.co; 3 Physician, M.Sc. Fundación Universitaria Autónoma de las Américas, Pereira, Colombia Email: freddy.barrios@uam,edu.co Fundación Universitaria Autónoma de las Américas Fundación Universitaria Autónoma de las Américas Pereira Colombia freddy.barrios@uam,edu.co; 4 Physician, M.Sc. Hospital Universitario San Jorge, Pereira, Colombia Email: oscar.suarez@uam.edu.co Hospital Universitario San Jorge Pereira Colombia oscar.suarez@uam.edu.co

**Keywords:** pneumonia, ventilator-associated, health education, dental, oral hygiene, intensive care units, nursing staff., neumonía asociada al ventilador, educación en salud dental, higiene bucal, unidades de cuidados intensivos, unidades de terapia intensiva, personal de enfermería., pneumonia associada à ventilação mecânica, educação em saúde bucal, higiene dentária, unidade de terapia intensiva, recursos humanos de enfermagem.

## Abstract

**Objective:**

This work sought to evaluate the impact of an educational intervention on oral hygiene care aimed at nursing care staff, on the incidence of Ventilator-Associated Pneumonia (VAP) in adults from an ICU in Colombia.

**Methods:**

Quasi-experimental study pre- and post-educational intervention aimed at nursing staff in which theoretical-practical sessions were conducted during 12 weeks to explain different oral hygiene techniques according to the oral conditions of patients. The study gathered sociodemographic, clinical, and characteristic variables of the oral and dental care received. The VAP was diagnosed according with international criteria.

**Results:**

The educational intervention received participation from 60 individuals (40 nurses and 20 nursing aides), 80% were women. The work collected data from 171 patients, 70 (40.9%) cared for after the educational intervention. Daily oral and dental care by the staff increased from 29.6% to 92.8% after the intervention. Although the accumulated incidence of VAP diminished from 8.9% to 2.8% and the rate of incidence dropped from 9 to 3.5 cases per 1,000 days of intubation, these changes were not statistically significant.

**Conclusion:**

The educational intervention aimed at the nursing staff in oral care reduced the incidence of VAP in adults connected to ventilator in ICU; although this decrease was not statistically significant, it was a clinically relevant result for the institution, which is why it is necessary to continue the educational strategies on oral health studied in this staff.

## Resumen

**Objetivo:**

Evaluar el impacto de una intervención educativa sobre los cuidados de higiene oral dirigida a personal asistencial de enfermería, en la incidencia de Neumonia Asociada a Ventilador (NAV) en adultos de una UCI en un hospital de Pereira, Colombia.

**Métodos:**

Estudio cuasiexperimental pre y posintervención educativa dirigida a personal de enfermería en la que se realizaron sesiones teórico-prácticas durante 12 semanas para explicar diferentes técnicas de higiene oral según la condición bucal de los pacientes. Se recolectaron variables sociodemográficas, clínicas y características de la atención bucodental recibida. Se diagnosticó la NAV de acuerdo con criterios internacionales.

**Resultados:**

En la intervención educativa participaron 60 personas (40 enfermeros y 20 auxiliares de enfermería), 80% eran mujeres. Se recolectaron datos de 171 pacientes, 70 (40.9%) atendidos después de la intervención educativa. La atención bucodental diaria por parte del personal se incrementó de 29.6% a 92.8%, después de la intervención. Aunque la incidencia acumulada de NAV disminuyó de 8.9% a 2.8% y la tasa de incidencia bajó de 9 a 3.5 casos por 1000 días de intubación, estos cambios no fueron estadísticamente significantes.

**Conclusión:**

La intervención educativa dirigida al personal de enfermería en cuidado oral redujo la incidencia de NAV en los adultos conectados a ventilador en UCI; si bien esta disminución no fue estadísticamente significativa, sí fue un resultado clínicamente relevante para la institución, por lo que deben continuarse las estrategias educativas estudiadas en salud bucal.

## Resumo

**Objetivo:**

Avaliar o impacto de uma intervenção educacional sobre cuidados de higiene bucal dirigida a profissionais de enfermagem sobre a incidência de Pneumonia Associada à Ventilação (PAV) em adultos ventilados em uma UTI na Colômbia.

**Métodos:**

Estudo quase experimental antes e após intervenção educativa dirigido a trabalhadores de enfermagem em que foram realizadas sessões teórico-práticas durante 12 semanas para explicar as diferentes técnicas de higiene bucal de acordo com a condição bucal dos pacientes. Foram coletadas variáveis ​​sociodemográficas, clínicas e características dos cuidados bucais recebidos. A PAV foi diagnosticada de acordo com critérios internacionais.

**Resultados:**

Na intervenção educativa, participaram 60 pessoas (40 enfermeiras e 20 auxiliares de enfermagem), 80% eram mulheres. Os dados foram coletados de 171 pacientes, 70 (40.9%) compareceram após a intervenção educativa. A higiene bucal diária pela equipe aumentou de 29,6% para 92.8% após a intervenção. Embora a incidência cumulativa de VAP tenha diminuído de 8.9% para 2.8% e a taxa de incidência tenha diminuído de 9 para 3,5 casos por 1000 dias de intubação, essas alterações não foram estatisticamente significativas.

**Conclusão:**

A intervenção educativa direcionada à equipe de enfermagem em higiene bucal reduziu a incidência de PAV em adultos conectados a um ventilador na UTI; embora essa redução não tenha sido estatisticamente significativa, foi um resultado clinicamente relevante para a instituição, portanto, as estratégias educacionais em saúde bucal estudadas nessas pessoas devem ser mantidas.

## Introduction

Mechanical ventilation-associated pneumonia (VAP) is one of the infections associated with health care most frequent in intensive care units (ICU) globally, with an incidence rate of 16.8 cases per 1000 ventilator-days, prolongs the hospital stay, increases care costs, and presents mortality up to 50%(1,2) caused by the migration of oropharyngeal microorganisms onto the pulmonary parenchyma and it is considered early if it develops during the first four days of ventilation, and late from the fifth day to 48 hours post-extubation.(^1^^,^^3^^)^ In Latin American countries, like Colombia, Ecuador, Venezuela, Mexico, Peru, and Bolivia, VAP has an incidence between 40% and 63%;(4) Cuba has been reported up to 70% of patients ventilated with VAP and mortality up to 60%.(5) Colombia estimates an occurrence of 60%, an incidence rate of 10 to 13.6 cases per 1000 ventilator-days and mortality of 70%.(6)

The World Health Organization has highlighted that VAP prevention measures are simple, low cost, and their effectiveness is based on the practices, responsibility and behavioral changes of the ICU health staff.(7) It has been demonstrated that greater occurrence of VAP is due to inadequate training of the health staff in elementary practices of infection prevention and control.(4) The Zero-VAP European Program has managed to reduce 43% of VAP and avoid 341 deaths annually. The most-efficient VAP prevention measures include oral care, given that it diminishes bacterial colonization of the pulmonary parenchyma. Eradication of microorganisms from the oral cavity, through mechanical hygiene (three times per day) and use of chlorhexidine in concentrations from 0.12% to 2%, reduce the risk of VAP.(8) Even in European countries, like Finland, 92% of the health staff had doubts on the indications of oral care.(9) Oral care is inconsistent and inadequate in ICU, according that found in the literature. Due to the foregoing, greater investment is required in VAP prevention programs, with enhancing of knowledge to modify the practices of the nursing staff regarding oral care, becoming a strategy that can be carried out in collaboration networks with other hospitals.

Given the lack of evaluations of these types of educational interventions on oral health in the practices by the health staff or on the incidence of VAP of critical patients in Colombia, this research work evaluated the impact of an educational intervention aimed at nursing staff in oral hygiene care on the incidence of VAP in adults treated with mechanical ventilation in an ICU in Pereira, Colombia.

## Methods

Quasi-experimental quantitative study to compare the incidence of pneumonia before and after implementing a care strategy of oral health with nursing staff. It included nurses from ICU and adult patients (> 18 years of age) in ICU with mechanical ventilation, who accepted to participate (or their relative or caretaker); the study excluded patients with any pulmonary infection as cause of admission to ICU. The first period (April 2 to September 22 of 2018) evaluated a group of patients seen under standard care.

An educational intervention was designed aimed at the nursing staff (40 professionals and 20 aides) that provides care to ICU patients, this had three stages: diagnostic, intervention, and evaluation. The diagnostic stage applied a questionnaire to the health staff to identify knowledge on oral care of patients in ICU, bearing in mind the standard care of patients evaluated during the first period.

From the results evidenced, the intervention stage was developed with theoretical base on the Keller's ARCS Motivational Design Model focused on maximizing the educator’s effectiveness by focusing the learner’s attention, relevance, trust and satisfaction via workshops, demonstrations, teaching aids, work models (10) with theoretical-practical sessions during 12 weeks, which explained different techniques of oral hygiene according to patient’s oral condition. The sessions addressed themes regarding the anatomy and physiology of the oral cavity, oral pathologies common in ICU patients, identification model of oral problems, ventilator-associated pneumonia, and oral hygiene techniques for intubated patients focused on the use of the toothbrush and dental floss to remove bacterial plaque, application of chlorhexidine with gauze to clean the teeth and all the mouth structures, the change of tube clamping to avoid ulcers, and the importance of hydrating the lips. Each of the participants individually carried out a supervised practice of oral care on a patient and supplies were delivered to the hospital to care for all patients admitted to the ICU until the end of the study. These practice sessions lasted for three months.

The evaluation stage conducted follow up of the nursing staff three times per week, identifying the use of these supplies and application of oral health in the patients. In addition, follow up was conducted of the hygiene activity records in the clinical charts. A second group of patients was evaluated (June 26 to December 27 of 2019). During the implementation of the care measure, the study had toothbrushes, tooth paste, and chlorhexidine at 0.12% to perform oral care of patients. In both groups, information was collected on the following variables: sociodemographic factors, clinical antecedents, remitting service, body mass index, oxygenation level on admission to ICU, renal function, and the characteristics of the oral and dental care received. As primary outcome, it was possible to register the presence of VAP and, as variables of interest, the orotracheal intubation time, stay in ICU, hospital stay, and hospital mortality were described.

The VAP diagnosis used combined criteria from: a) the American Thoracic Society (ATS) together with the Infectious Diseases Society of America (IDSA), and b) Centers for Disease Control (CDC), included in the 2013 Colombian consensus on nosocomial pneumonia (11) and accepted in Colombia by the National Health Institute and the Ministry of Health in its 2017 update of the National Epidemiological Surveillance Protocol for Device-Associated Infections.(3) According with the aforementioned, the study considered as case every pneumonia documented in the unit 48 h after endotracheal intubation was established in the presence of new or progressive infiltrates and persistent in the chest X-ray (or cavitation or consolidation), in addition to at least one of the following criteria: fever (> 38 °C) or hypothermia with no other recognized cause, leukopenia (< 4000 PMN/ml) or leukocytosis (> 12000 PMN/ml), altered mental state with no other recognized cause in adults > 70 years of age, purulent tracheal secretions, and oxygenation disorder. All the cases were confirmed by clinic and none by laboratory and, finally, were classified as early or late VAP cases.(12)

Bivariate analysis was performed according to the pre- and post-intervention group; proportions were compared through the Chi-squared test. When its assumptions were not fulfilled, Fisher's Exact test was used. Medians and interquartile ranges were compared via the Mann-Whitney U test, upon applying the Shapiro-Wilks test to verify the fit to the normal distribution. For the multivariate analysis, the VAP rate was constructed according to the days of intubation. With significant variables identified in the bivariate analysis, Poisson’s regression model was constructed to adjust the estimated effect of the intervention by potential confounders. Incidence rate ratios (IRR) were estimated as measures of association with 95% confidence intervals and p values. RStudio software, version 1.2.5033 (©2009-2019 RStudio, Inc.) was used.

The project was supported by the ethics committee at Fundación Universitaria Autónoma de las Américas and by the research committee at the health institution where the research took place.

## Results

In the stages of the educational intervention, 40 nursing professionals and 20 nursing aides participated with work experience in ICU between 2 and 10 years and 80% were women. The initial questionnaire evidenced that all the staff had knowledge about VAP and with respect to the patient’s oral care, 16% knew of chlorhexidine for oral use, 5% revised the patient’s oral and dental state and evolution, 93% was unaware of the most frequent oral pathologies in intubated patients and their prevention.

Data was collected from 171 patients, 70 (40.9%) of them seen after conducting the educational intervention. In total, 54% of the participants were men, with a mean age of 56 years for both groups; prevalence of patients affiliated to the subsidized scheme, without statistically significant differences. In the group intervened, higher prevalence was found of cardiopathy (*p* = 0.02), greater proportion of individual’s body mass index classified as normal (*p* = 0.06), greater PAFI median (*p* = 0.02), and greater use of phenytoin (*p*=0.007); moreover, lower frequency of high BUN was identified (*p* = 0.007). The other characteristics are described in Table 1.

**Table 1:** Medical antecedents, characteristics of admission and care of patients with mechanical ventilation in ICU of a tier III hospital

Characteristic	**Not intervened (*n* = 101)**	**Intervened (*n* = 70)**	*p value*	Total (*n* = 171)
Mean age (IQR)	56 (39-72)	56 (40-70)	0.645	56 (39-70)
Age groups; *n* (%)			0.812	
< 20 years	4 (3.9)	3 (4.3)		7 (4.1)
20 to 39 years	23 (22.7)	13 (18.5)		36 (21.1)
40 to 64 years	35 (34.6)	29 (41.4)		64 (37.4)
65 years or more	39 (38.6)	25 (35.7)		64 (37.4)
Women; *n* (%)	41 (40.6)	30 (42.8)	0.768	71 (45.5)
Social security; *n* (%)				
Subsidized	74 (73.3)	48 (68.6)	0.779	122 (71.3)
Traffic accident policies	13 (12.9)	10 (14.3)		23 (13.5)
Contributive	11 (10.9)	7 (10)		18 (10.5)
Other	3 (2.8)	5 (7.1)		8 (4.7)
Antecedents; *n* (%)				
Diabetes Mellitus	15 (14.8)	8 (11.4)	0.519	23 (13.4)
Arterial hypertension	28 (28.1)	-		28 (-)
Chronic kidney disease	14 (13.8)	5 (7.1)	0.169	19 (11.1)
COPD	12 (11.8)	7 (10)	0.700	19 (11.1)
Cardiopathy	6 (6.9)	13 (18.6)	0.020	20 (11.7)
Asthma	1 (0.9)	0 (0)	0.591	1 (0.6)
Antecedents; *n* (%)	15 (14.8)	8 (11.4)	0.519	23 (13.4)
Infectious antecedents; *n* (%)				
HIV (AIDS)	3 (2.9)	0 (0)	0.270	3 (1.7)
Pulmonary tuberculosis	5 (4.9)	3 (4.3)	0.573	8 (4.7)
Urinary tract infection				1 (1.0)
Referral service				
Emergency	80 (79.2)	57 (81.4)	0.122	137(80.1)
Surgery	1 (0.9)	4 (5.7)		5 (2.9)
Hospitalization	20 (19.8)	9 (12.8)		29 (16.9)
BMI classification; *n* (%)			0.063	
Low weight	10 (9.9)	2 (2.8)		12 (7.0)
Normal	50 (49.5)	48 (68.6)		98 (57.3)
Overweight	34 (33.6)	16 (22.8)		50 (29.2)
Obesity	7 (6.9)	4 (5.7)		11 (6.4)
State of oxygenation; *n* (%)				
Normal (400-500)	23 (22.7)	8 (11.4)	0.138	31 (18.1)
Mild hypoxemia (300-399)	15 (14.8)	7 (10.0)		22 (12.9)
Moderate hypoxemia (200-299)	28 (27.7)	22 (31.4)		50 (29.2)
Severe hypoxemia (< 199)	35 (34.6)	33 (47.1)		68 (39.7)
PAFI median (IQR)	260 (158 -386)	215 (138-286)	0.029	239 (149-348)
Kidney function on admission; *n* (%)				
Creatinine (≥ 1 .5 mg/dL)	33 (32.7)	23 (32.8)	0.980	56 (32.7)
Ureic nitrogen (≥ 38 .6 mg/dL)	60 (59.4)	27 (38.6)	0.007	87 (50.8)
Systemic medication; *n* (%)				
Phenytoin	26 (25.7)	32 (45.7)	0.007	58 (33.9)
Corticoids	1 (0.9)	1 (1.4)	0.793	2 (1.2)
Nutritional support; *n* (%)				
Enteral	83 (82.2)	64 (91.4)	0.172	147 (85.9)
Parenteral	15 (14.8)	4 (5.7)		19 (11.1)
None	3 (2.9)	2 (2.8)		5 (2.9)

IQR= Interquartile range. COPD= chronic obstructive pulmonary disease. BMI= body mass index. PAFI= ratio of PaO/fraction of inspired oxygen.

The record of activities with oral care rose from 29.6% to 92.8%, tooth brushing by patients was registered in 45.6% and application of chlorhexidine in 70% of patients seen after the intervention, with significant differences (p < 0.001) compared with patients before the intervention. Table 2 presents the variables referring to oral and dental care, like frequency of tooth brushing and use of chlorhexidine.

**Table 2:** Characteristics of oral care of patients with mechanical ventilation in ICU of a tier III hospital

Characteristic	**Not intervened (*n* = 101)**	**Intervened (*n* = 70)**	*p value*	Total (*n* = 171)
Oral and dental care			<0.001	
None	71 (70.3)	5 (7.1)		76 (44.4)
One time	25 (24.7)	54 (77.1)		79 (46.2)
Two or more times	4 (4.9)	11 (15.7)		16 (9.35)
Tooth brushing	1 (0.9)	34 (48.6)	<0.001	35 (20.5)
Chlorhexidine - Mouthwash	3 (2.9)	49 (70)	<0.001	52 (30.4)
Aides participate in caring	33 (32.7)	66 (94.3)	<0.001	72 (42.1)
Daily aspiration of secretions			<0.001	
One time	0 (0)	2 (2.8)		2 (1.2)
Two times	64 (63.4)	63 (90		127 (74.3)
Three times	31 (30.7)	5 (7.1)		36 (21)
Four times	6 (5.9)	0 (0)		6 (3.5)

Table 3 shows the description of other variables of interest and Table 4 details the results of the multivariate analysis. The accumulated VAP incidence diminished from 8.9% to 2.8% after the intervention and the rate dropped from 9 to 3.5 cases per 1000 days of intubation, both differences were not significant. The work estimated a decrease in mortality from 38.6% to 30% after the intervention, without statistical significance. All the VAP cases were classified as late (Table 3).

**Table 3:** Outcomes of patients with mechanical ventilation in ICU of a tier III hospital

Characteristic	**Not intervened (*n* = 101)**	Intervened (*n* = 70)	*p-value*	Total (*n* = 171)
Ventilator-associated pneumonia	9 (8.9%)	2 (2.8%)	0.203	11 (6.4%)
Median days of intubation (IQR)	7 (4-13)	7 (4-12)	0.383	7 (4-13)
Density of incidence	9.0/1,000 days	3.5/1,000 days	0.225	7.0 /1,000 days
Median ICU stay (IQR)	10 (5-17)	10 (6-16)	0.755	10 (5-16)
Median Hospital stay (IQR)	17.5 (9.5-28.5)	18 (13-29)	0.704	18 (11-29)
Deceased	39 (38.6%)	21 (30%)	0.246	60 (35.1%)

IQR= Interquartile range. ICU= Intensive care unit

The multivariate model constructed with Poisson’s regression permits estimating 65% reduction of the incidence rate after the intervention adjusted by the referral service, antecedent of cardiopathy or chronic kidney disease, body mass index, PAFI classification, and ureic nitrogen (>20 mg/dL). This effect found showed no confidence intervals or statistically significant probability values.

**Table 4:** Poisson regression model for incidence rate ratio of ventilator-associated pneumonia in patients with mechanical ventilation in ICU of a tier III hospita**l**

Characteristic	Raw IRR (95% CI)	***p* value**	Adjusted IRR (95% CI)	***p-*value**
Intervention in oral care	0.38 (0.04-1.8)	0.225	0.35 (0.07-1.8)	0.213
Referral from				
Hospitalization	4.8 (0.1-37.6)	0.2280.470-	6.4 (0.58-71.5)	0.128
Surgery	1.6 (0.27-7.2)		1.5 (0.37-6.6)	0.534
Emergency	1		1	
Antecedent of cardiopathy	0.64 (0.14-4.5)	0.7560.198	0.45 (0.04-4.3)	0.496
Antecedent of kidney disease	2.5 (0.44-10.6)		2.1 (0.4-9.6)	0.326
BMI				
Low weight	0 (0-4.1)	0.339-	0.05 (0 -.)	0.995
Normal	1	-	1	-
Overweight or obesity	0.77 (0.16-3)	0.701	0.55 (0.15-2.02)	0.372
State of PAFI oxygenation				
Normal	1	-	1	-
Mild	1.58 (0.11-21)	0.665	1.1 (0.15-8.5)	0.905
Moderate	0.79 (0.09-9.5)	0.797	0.6 (0.1-4.2)	0.670
Severe	0.83 (0.11-9.1	0.809	0.5 (0.07-3.1)	0.452
Ureic nitrogen > 20 mg/dL	2.1 (0.51-12.4)	0.271	2 (0.4-9.2)	0.340

IRR: incidence rate ratio; BMI: body mass index; mmHg: millimeters of mercury; PAFI= ratio of PaO/fraction of inspired oxygen. Pseudo R2 = 0.139 (*p* = 0.488)

## Discussion

Within the national context, and according with the literature reviewed, this is the first work that seeks to verify the impact of an educational intervention in the nursing staff of adult ICU on oral and dental hygiene measures to reduce VAP in patients treated with invasive mechanical ventilation. In this sense, the contribution of this research lies in that its results constitute an important input for the reference institution to generate strategies based on local evidence that permit reducing this adverse event associated with health care, being a care priority according with the guidelines by the Ministry of Health and NHI on the quality and safety of care, especially regarding device-associated infections.(3) Although in the group of patients who received care from the trained nursing staff no statistically significant reduction was shown of the VAP incidence or VAP rate per ventilator days, this reduction is clinically relevant for the clinical practice, given that it allows the institution to make decisions to continue improvement processes of interventions in oral and dental hygiene in critically-ill patients with high risk of VAP. Thus, the fact of reducing the incidence from 8.9% to 2.8% and from 9 to 3.5 cases/1000 ventilator days evidences an important reduction, considering the severity of a single case of VAP, in terms of hospital stay, use of antibiotics, medical supplies, and other costs associated with care that a public hospital must destine to caring for events related with providing health care.(13)

Likewise, the unadjusted decrease in overall mortality in the unit from 38.6% to 30%, post-intervention, is a valuable data for the institution, given that - although not statistically significant - it does provide insights on the impact the strategy could have in the long term, besides providing tools for future studies to evaluate the other clinical factors and factors derived from care that impact on the reduction or increase in mortality rates in the service. 

Of the predictors included in the equation, with a clinically relevant effect (although not statistically significant), it is possible to affirm that - for the group of patients benefiting from the training intervention in oral and dental hygiene to the nursing staff (post group) - this intervention managed to reduce the risk VAP up to 65% compared with patients from the group not exposed (pre group). In addition, patients from hospitalization and surgery had higher risk of VAP (up to 5.4 times and 0.5 times more, respectively, compared with those admitted from emergency). Similarly, patients with antecedent of kidney disease also had twice the risk of developing VAP with respect to patients without this antecedent. Besides, patients with registry of ureic nitrogen > 20 mg/dL had twice the risk of VAP when compared with those who had kidney function above said value. All these associations were produced by adjusting by the effect of the other co-variables included in the final equation and with these constants remaining, and although none of them was statistically significant, they were clinically relevant.

Our study did not find that antecedent of cardiopathy, overweight or obesity, oxygenation measured via PAFI (mmHg/FIO_2_), and the value of ureic nitrogen were predictors associated with VAP incidence in the study population. However, in spite of their non-significance, these variables were included in the final Poisson regression model for adjustment effects as possible confounding variables. Additionally, these were left in the final equation due to the model´s convergence.(14) Thus, the apparently not significant, paradoxical results and contradictory with the literature, seen in Table 4 (antecedent of cardiopathy, overweight or obesity, moderate and severe PAFI, as protective factors and which would reduce VAP incidence) lack clinical interest for the effects of this study. Further, even when several authors(15,16) have demonstrated that some of these conditions are risk factors, their results are not comparable with those in this study, given that, in addition to the p value of association, the risk measurements employed in said investigations (17) have included Odds Ratio, Hazard Ratio, and Relative Risk, but not the IRR that is used in our study, given that the dependent variable of interest herein is the VAP measured as a rate of incidence.

In this study, the initial rate of VAP prior to the intervention was nine cases per 1000 days of intubation, lower than in various countries in the world, as indicated by the report by the International Nosocomial Infection Control Consortium, (1) where the global rate was 16.8 per 1000 days in 503 ICU from 43 participating countries. It was also lower than the rate reported in the European study denominated ICU-HELICS (Hospitals in Europe Link for Infection Control through Surveillance) (18,19) in which the indicator varied from 9.9 pneumonia cases/1,000 dMV (Germany) to 24.5 (Holland); and lower than in Spain, which reported a VAP rate that ranges between 15.5 and 17.5 episodes/1,000 dMV, with mortality varying between 30% and 34.8%, according with records from the National Study on Nosocomial Infection Surveillance in ICU (ENVIN-UCI, for the term in Spanish), conducted from 2003 to 2005 and which included over 21000 patients.(20) However, our result was higher than that reported in 1991 in the United States by the National Nosocomial Infections Surveillance System (NNIS): 5.8 cases/1000 days of MV; (17) although it should be clarified that these differences may be because during said moment, the VAP diagnostic criteria had not been updated.

When comparing within the Latin American context, our rates were lower than those reported by Mexico (18.6 cases per 1000 days),(4) among others. In relation with the VAP incidence, our baseline proportion was 8.9%, lower than in countries, like Cuba, which have reported incidences of up to 70% of patients with invasive mechanical ventilation.(19)

Regarding the comparison with Colombia, the data are relatively lower than in the rest of the national studies, with prevalence reported up to 60% and rates that range between 10 and 13.6 cases per 1000 ventilator-days, with mortality at 70%. Our VAP prevalence in the pre-intervention group was of 8.9%, lower than in similar high-complexity hospitals, like that reported in Cúcuta, during the period between 2013 and 2016, during which data was analyzed from 69 patients with inclusion conditions similar to this study, finding that, with the same diagnostic criteria, late VAP presence was of 42%.(7)

Prevalence of VAP was also lower than in the study by Ortiz *et al.*, who conducted a prospective cohort study in 39 ICU from eight cities in Colombia, from 2007 to 2009, identifying and classifying VAP according to CDC criteria.(20) That study included 31622 patients, in which were diagnose 1944 episodes of device-associated infection, of which 858 corresponded to VAP (44.1%); this multicenter study found a rate of 7.4 cases per 1000 days/ventilator for 2010.

As limitations to identify an effect with statistical significance, it should be noted that reduced sample size could affect the complete and ideal convergence of the final model, as well as the regression coefficients and, hence, the association measurement (IRR) calculated via Poisson’s regression.(21) Besides, the VAP incidence was relatively low *per se*, therefore, there may be residual variance not explained by the effect of the variables included in the model. As limitations of the study, the following are recognized: a) the failure to isolate the germs through cultures of tracheal secretions, which did not allow comparing the microbiota distribution of early and late VAP, as done in other studies in the country, b) the limited sample size that subtracts statistical significance from the findings, and c) it was not possible to verify on the field all the variables, beyond the clinical record especially of care and oral health characteristics by the nursing staff. It is possible that observations by the staff condition the effort of conducting adequate care, but without this supervision the mere record does not transcend (Hawthorne effect)**.** All these biases could have been controlled through a clinical trial, however, due to ethical considerations that did not allow randomization of patients nor allocation of treatment arms, there were no feasibility conditions to carry out this type of study.

It should be highlighted that the findings herein are complemented with observations during the field work phase, which evidenced commitment by the nursing staff during the strategy implementation phase, who attended all training sessions and the results demonstrate they placed into practice, during the study execution period, everything learnt during said training. This information is also relevant, to the extent that it evidences existence of the commitment by the staff and disposition to carry out health interventions derived from epidemiological studies, besides demonstrating that it is even possible to carry out experimental studies or with other types of designs in the future in the hospital, where collaboration from the care staff is fundamental.

Regarding differences in the conditions of both groups, some of these were statistically significant in the bivariate analysis between the pre and post groups, also possibly explaining, besides the effect of the intervention evaluated, reduction of VAP between one and another group. Thus, our findings agree with Hairani *et al*.,(22) who demonstrated that inadequate acute kidney function (included in the SOFA score) is associated with prolonged intubation time and with VAP (*p* < 0.01). Younan *et al*.,(23) also confirmed this association (*p* < 0.001), although of greater relevance within the context of traumatized patients.

Furthermore, variables related with oral care occurred with greater prevalence in the group intervened: oral and dental care one or more times per day, increased tooth brushing, and use of chlorhexidine and oral antiseptics, as well the increase of nursing staff that participate in caring and increase in the number of daily aspirations of secretions. As in a quasi-experimental study conducted with 71 nurses in Malaysia, which compared knowledge and activities destined for prevention before and after receiving training on VAP (before the intervention: 63.17 ± 9.34; after the intervention: 95.99 ± 4.68; *p* < 0.001) and which compared the incidence of VAP, before 22 cases from 101 patients and after seven cases from 110 patients, evidencing that training of the health staff in oral care influences in increasing oral care practices in patients and managing to reduce VAP incidence.(24)

Educational programs addressing oral care can improve the quality of the care provided by the nursing staff; the American Association of Critical Care Nurses states that oral health is a basic requisite of the nursing practice, which is why it is necessary to implement protocols that guide care, as well as training programs.(25) Prevention interventions in oral health must be constant in ICU, bearing in mind that these contribute to diminishing inflammatory, infectious, and painful problems. Odontologists must be part of this multidisciplinary team with adaptation of treatments and guidance to the health staff. Thus, it becomes necessary to implement educational and adaptation actions aimed at the nursing staff, to provide higher quality and professional integrity with critical patients, as well as humanization of actions aimed at promoting oral health.

It is recommended to implement continuous training interventions in nursing care aimed at reducing complications and increasing the quality of care, construct guides and protocols with packages of VAP prevention measures, besides using follow-up tools that will permit evaluating the care procedures. The work highlights the need to generate multidisciplinary actions applied to the daily practice, with participation by the odontologist in hospital settings, evaluation, and critical patient care, aimed at the diagnosis and protocolized treatment of oral pathology, as well as support to the care staff, which contributes with adapting treatments and guiding oral care.

## Conclusion

The intervention seems useful (reduces incidence rate up to 65% when adjusting for multiple co-factors); however, no statistical evidence supports this finding and evidence exists that the staff that conducts oral care in patients is highly adherent to recommendations related with the intervention proposed
